# Mitochondrial injury and complement dysregulation are drivers of pathological inflammation in viral myocarditis

**DOI:** 10.1128/jvi.01804-24

**Published:** 2025-01-23

**Authors:** Yasir Mohamud, Amirhossein Bahreyni, Sinwoo Wendy Hwang, Jingfei Carly Lin, Zhihan Claire Wang, Jingchun Zhang, Honglin Luo

**Affiliations:** 1 Centre for Heart Lung Innovation, St. Paul’s Hospital 8156 , Vancouver, British Columbia, Canada; 2 Department of Pathology and Laboratory Medicine, University of British Columbia 8166 , Vancouver, British Columbia, Canada; University of North Carolina at Chapel Hill, Chapel Hill, North Carolina, USA

**Keywords:** myocarditis, coxsackievirus b3, complement, mitochondria, heart failure, cd55, cd59, inflammation

## Abstract

Enteroviruses cause nearly 1 billion global infections annually and are associated with a diverse array of human illnesses. Among these, myocarditis and the resulting chronic inflammation have been recognized as major contributing factors to virus-induced heart failure. Despite our growing understanding, very limited therapeutic strategies have been developed to address the pathological consequences of virus-induced chronic innate immune activation. Coxsackievirus B3 (CVB3) was used as a model cardiotropic enterovirus. We leveraged *in vitro* cell-based studies to investigate cardiomyocyte and macrophage interaction during CVB3 infection, as well as animal studies and unique human cardio specimens to evaluate mechanisms of viral heart injury. We present evidence that viral myocarditis is in part exacerbated by pathological activation of the complement pathway in cells, mice, and human cardiac tissues. We demonstrate unique cell type-specific responses to viral infection that are exacerbated by mitochondrial injury in cardiomyocytes and NFκB-dependent pro-inflammatory response in macrophages. Macrophages are robustly activated by damage-associated mitochondrial components, including mitochondrial proteins and lipid extracts. Mechanistically, we identify complement protective factors CD59/protectin and CD55/DAF as novel targets of viral proteinase that acts to release the brakes on complement-mediated autoinjury. Collectively, our study highlights a novel mechanism that can act as a potential contributor to CVB3 pathogenesis through mitochondrial injury-mediated autoimmunity.

**IMPORTANCE:**

This study sheds light on how enteroviruses, specifically coxsackievirus B3, may contribute to heart failure by triggering harmful immune responses in the heart. We discovered that viral infections in heart cells cause mitochondrial damage, which in turn activates a destructive immune response involving the complement system. This immune activation is one of the significant contributors that lead to further injury of heart tissues, worsening the damage caused by the virus. Additionally, we identified key protective molecules that are targeted and disrupted by the virus, allowing the immune system to attack the heart even more aggressively. Understanding these mechanisms may provide additional insights into how viral infections can lead to chronic heart conditions and suggests potential therapeutic targets to prevent or reduce heart damage in patients affected by viral myocarditis.

## INTRODUCTION

Viral myocarditis, a condition characterized by the inflammation of the heart muscle, poses significant health challenges and has been a topic of increased interest in recent years. This condition unfolds in three distinct phases: an acute phase characterized by intense viral infection, a sub-acute stage predominantly driven by pro-inflammatory autoimmunity, and the chronic stage (>2 weeks) associated with either resolution or heart failure (1–3). Although it is difficult to pinpoint the exact percentage of viral myocarditis cases that progress to heart failure, factors such as the severity of myocarditis, the specific virus involved, and the individual’s overall health are crucial in determining the outcome. Indeed, a percentage of cases may resolve without severe long-term effects, while others can progress to heart failure (1, 2). Indeed, viral myocarditis can be triggered by various viruses, including parvovirus, adenovirus, coronaviruses, and enteroviruses, with coxsackievirus B3 (CVB3) serving as a well-established research model (1). Notably, the broad-spectrum anti-inflammatory therapies traditionally used in clinical settings have not shown substantial benefits in treating this condition (4). Recent efforts are attempting to identify specific inflammatory pathways that can be directly targeted to minimize autoimmune bystander injury to cardiac tissue (1).

The complexity of viral myocarditis lies in its unique inflammatory signatures, which, if not identified and managed properly, can exacerbate the disease. An ideal response to such an infection should involve the activation of specific pathways that combat the infection while minimizing harm to the host (5). However, deviation from this balanced response, such as the activation of pathological inflammation with excessive bystander injury, can lead to severe consequences, including heart failure (1, 5, 6).

Viral myocarditis is characterized by a multifaceted inflammatory response that begins with direct viral injury to the heart muscle, followed by secondary damage mediated by the host’s immune system (1–3). The initial defense involves the innate immune system, which activates cellular responses such as macrophages and dendritic cells, as well as humoral responses such as complement to combat the viral infection. Additionally, the adaptive immune response plays a critical role in orchestrating a more specific and sustained defense against pathogens (6).

In our current research, we have identified viral-mediated mitochondrial injury as a pathological driver of innate immune activation through diverse approaches that include macrophage pro-inflammatory signaling, complement activation, and deregulation, ultimately converging in viral heart failure. Although traditionally linked to anti-bacterial and anti-viral defenses (7), the complement pathway plays a significant role in viral heart injury (8). Our study reveals that the cardiotropic virus, CVB3, inflicts damage to mitochondria within cardiomyocytes. We found that components associated with damaged mitochondria, such as mitochondrial lipids and proteins, are capable of inducing pro-inflammatory macrophage activation independently of viral replication. Additionally, our study has linked mitochondrial damage to pathological activation of the complement system. Lastly, we identify a novel mechanism by which CVB3 downregulates complement protective factors to further exacerbate the cardiac bystander injury.

## RESULTS

### CVB3-induced cardiomyocyte injury is associated with pro-inflammatory macrophage activation

**Fig 1:**
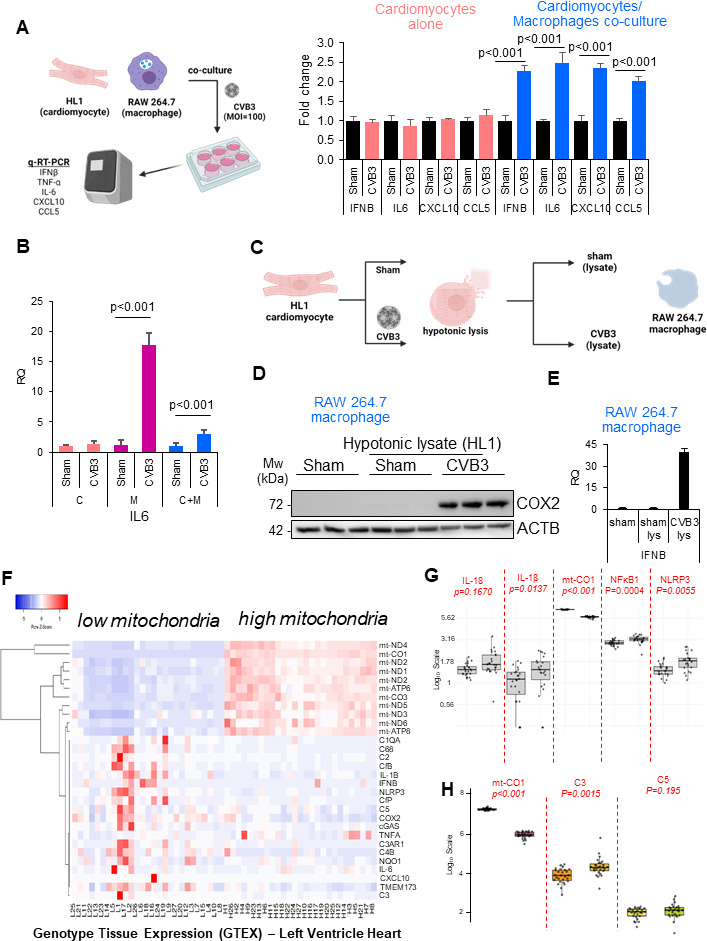
CVB3-induced cardiomyocyte injury is associated with pro-inflammatory macrophage activation. (**A**) Schematic representation of monoculture or co-culture experiments with murine HL-1 cardiomyocytes and murine RAW 264.7 macrophages. Cells were infected with CVB3 (multiplicity of infection [MOI] = 100, 48 h) and subsequently subjected to inflammatory gene expression analysis and presented as relative quantitation (RQ) (mean ± SD, *n* = 3). Statistical analysis was performed using analysis of variance with Tukey post hoc test. (**B**) HL-1 cardiomyocyte (C), RAW264.7 macrophages (M), and co-culture (C + M) were infected as above and subjected to RT- qPCR analysis for the target gene *Il6* and normalized to *Actb*. (**C**) Schematic illustration depicting sham- or CVB3-infected (MOI = 100, 8 h) HL1 cardiomyocytes subjected to hypotonic lysis. (**D and E**) Hypotonic lysate (**B**) was diluted in culture medium and incubated with RAW264.7 cells for 4 h, and cells were then subjected to Western blot analysis for macrophage activation marker, COX2 (**C**), and qPCR for anti-viral type I IFN gene (**D**). (**F–H**) Low mitochondrial gene expression is associated with increased inflammation in human left ventricle hearts, as shown by analysis of the Genotype Tissue Expression (GTEX) public data set (*n* = 54). Statistical analysis between low- and high-mitochondria groups was performed using an unpaired Student *t*-test.

In exploring the complex mechanisms underlying viral heart failure, it is evident that disease pathogenesis involves not only direct viral-mediated damage to cardiomyocytes but also significant secondary damage driven by inflammatory responses (1–3). Among the various pro-inflammatory cells, macrophages play a crucial role in responding to infections (9). CVB3 is an established model for studying viral myocarditis, largely due to the existence of effective animal models. We first evaluated CVB3-induced inflammatory gene expression in a co-culture system comprising HL1 cardiomyocytes and the murine macrophage cell line RAW264.7. HL1 cells in monoculture or co-culture with RAW264.7 macrophages were infected with CVB3 for 48 h. Surprisingly, no elevated inflammatory gene expression was detected in monocultures of HL1 cardiomyocytes compared to non-infected controls, whereas pooled RNA extracted from HL1 co-cultured with RAW264.7 cells demonstrated a twofold induction in several cytokines and chemokines, including *Ifnb*, *Il6*, *Cxcl10*, and *Ccl5* (Fig. 1A). Of note, monoculture of RAW264.7 macrophage cells treated with CVB3 was associated with significantly elevated *Il6* cytokine expression (Fig. 1B). To assess the dynamics of HL1 cardiomyocyte and RAW264.7 macrophages during infection, we performed a lysate transfer experiment from control or CVB3-infected HL1 cells (Fig. 1C). RAW264.7 cells were incubated with these respective lysates for 4 h and then subjected to Western blot analysis and real-time quantitative reverse transcription PCR (RT-qPCR). Interestingly, lysates from CVB3-infected HL1 cardiomyocytes but not sham-infected controls were able to induce COX2 expression (Fig. 1D), a marker of macrophage activation as well as *Ifnb* gene expression (Fig. 1E). Taken together, our data support a potential role for macrophage cells in the pro-inflammatory signaling during CVB3 infection.

We next sought to evaluate the mechanism behind macrophage activation following CVB3 infection. Given the abundance of mitochondria in cardiac tissue and the fact that mitochondrial dysfunction is a hallmark of heart failure (10, 11), we sought to explore whether changes in mitochondrial state could be correlated with pathological cardiac inflammation. Indeed, CVB3-infected human cardiac tissue demonstrated pathological alterations in monocyte infiltration as assessed by cyclic GMP-AMP synthase (cGAS)-positive staining (Fig. S1). Data from the Genotype Tissue Expression (GTEX) database were used to identify associations in gene expression in the left ventricle of human hearts from 54 samples. To assess the role of mitochondria, samples were first segmented into low-mitochondria and high-mitochondria groups (*n* = 27 per group) using the mitochondrial gene cytochrome c oxidase I (*mtCO1*) as a proxy marker of mitochondrial DNA (mtDNA) abundance and indirectly, mitochondrial biomass in the cardiac tissues of the GTEX database (Fig. 1F). Following this analysis, we observed clustering of samples in the low mitochondria segment that exhibited elevated expression of pro-inflammatory genes (Fig. 1F). Low *mtCO1* expression also correlated with elevated *IL-1β*, *NLRP3*, *NFkβ1*, and complement *C3* expression in cardiac tissue (Fig. 1G and H). Collectively, this evidence suggests that mitochondrial dysfunction may underlie the pathological inflammation present in the heart.

### Mitochondrial double-stranded DNA is released after CVB3 infection, but mtDNA depletion is not sufficient to block CVB3-mediated macrophage activation

**Fig 2:**
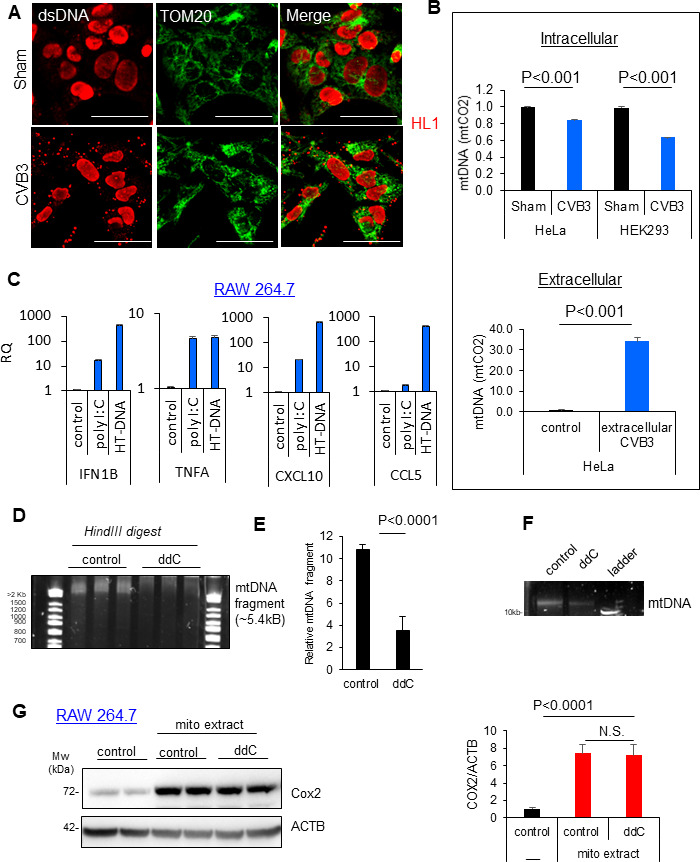
Mitochondrial dsDNA is released upon CVB3 infection, but mtDNA depletion alone is not sufficient to block CVB3-mediated macrophage activation. (**A**) HL-1 murine cardiomyocytes were sham or CVB3-infected (MOI = 100, 8 h), followed by confocal microscopy analysis for mtDNA release (cytosolic dsDNA) and the mitochondrial marker TOM20. Scale bar = 20 µm. (**B**) Extracellular mtDNA is increased following CVB3 infection. HeLa and HEK293 cells were infected with CVB3 (MOI = 10 and 100, respectively, 5 h) and then subjected to fractionation of cytosolic and mitochondrial compartments. Cytosolic mtDNA was assessed via qPCR analysis of the mitochondrial gene marker mtCO2 and normalized to total cell protein. Extracellular mtDNA was assessed in supernatants of control or CVB3-infected HeLa cells (MOI = 10, 5 h) using qPCR analysis of the mitochondrial gene marker mtCO2. Statistical analysis between sham and CVB3 groups was performed using an unpaired Student *t*-test. (**C**) qPCR assessment of inflammatory gene expression in RAW264.7 macrophages transfected with either poly I:C (1 µg/mL) or herring testes (HT)-DNA (3 µg/mL) (mean ± SD, *n* = 3). (**D**) Extracted mtDNA from control or ddC-treated HeLa cells (50 µg/mL for 144 h) was subjected to restriction enzyme digestion with HindIII and analyzed by acrylamide gel electrophoresis. (**E**) Density of mtDNA fragment post-HindIII digestion at 5.4 kB was quantified (mean ± SD, *n* = 3). (**F**) Undigested mtDNA was also analyzed by agarose gel electrophoresis. (**G**) RAW264.7 macrophages were treated with mitochondrial extracts from control or ddC-treated cells. Cell lysates were subjected to Western blot analysis for the macrophage activation marker COX2 and normalized to ACTB. Relative COX2 expression was quantified in the right bar plot. Statistical analysis was performed using analysis of variance with Tukey post hoc test.

To better investigate the mechanisms behind mitochondria-mediated inflammation in viral myocarditis, we studied CVB3 infection in a cell culture model. HL1 murine cardiomyocytes were infected with CVB3 (multiplicity of infection [MOI] = 100) or phosphate-buffered saline (PBS) as sham control for 8 h. Upon infection, we detected significant perturbations in the visible mitochondrial network with the appearance of TOM20+ fragmented mitochondrial puncta (Fig. 2A). Coincident with this, CVB3-infected HL1 cardiomyocytes displayed accumulation of double-stranded DNA (dsDNA) in cytosol, with intact nuclear staining, suggesting mtDNA leakage. Next, we inquired whether CVB3 infection could directly induce mtDNA release. First, CVB3-susceptible cell lines (HeLa and HEK293 cells) were infected with CVB3 for a duration of 5 h, consistent with the emergence of mitochondrial morphology changes and perturbations. Subsequently, cytosolic DNA was harvested from both sham and infected cells, as well as from respective culture media, and subjected to quantitative PCR analysis with specific mtDNA primers targeting the cytochrome c oxidase 2 (*mtCO2*) gene. Results from these experiments demonstrated a significant downregulation of mtDNA within CVB3-infected cells (Fig. 2B), coincident with an elevation of mtDNA levels in the extracellular supernatants (Fig. 2B). Mislocalized DNA is recognized in cells by the cytosolic DNA sensor cGAS (12). Interestingly, when the cGAS agonist herring testes-DNA was delivered directly into cells using a liposome-based transfection reagent, RAW264.7 macrophage cells demonstrated robust activation of cGAS-stimulator of interferon gene (STING) downstream inflammatory cytokines and chemokines (Fig. 2C), suggesting that endocytosed dsDNA can induce cGAS-STING-mediated inflammatory activation of macrophages. As expected, the double-stranded RNA (dsRNA) mimetic poly I:C also robustly activated inflammatory gene signaling (Fig. 2C). We next sought to address whether depletion of mtDNA could ameliorate the inflammatory activation of RAW264.7 macrophages. To test this, we utilized ddC, a nucleoside analog which has previously been shown to robustly deplete mtDNA (13). To evaluate the efficacy of ddC treatment, mtDNA was isolated from control and ddC-treated cells, then subjected to acrylamide gel electrophoresis following restriction enzyme digestion. Results demonstrated a significant reduction (~70%) in an expected mtDNA fragment above the 2 kb ladder post-HindIII digestion (Fig. 2D and E) as well as full-length mtDNA plasmids (Fig. 2F) following ddC treatment. We next sought to test the effects of mtDNA depletion on mitochondria-mediated macrophage activation. Mitochondria were harvested using an optimized mitochondria isolation protocol that leverages a recombinant-tagged mitochondrial outer membrane protein (TOM20-Flag) and anti-flag-based magnetic bead isolation (Fig. S2). RAW264.7 macrophages were treated with mitochondrial extracts from control or ddC-treated cells. Interestingly, both control and ddC-treated mitochondria robustly activated RAW264.7 macrophages (Fig. 2G). Together, this evidence suggests that mtDNA depletion alone is not sufficient to block macrophage activation, indicating that additional components, such as proteins and lipids, within damaged mitochondria are required for triggering macrophage activation.

### Mitochondrial proteins and lipids are sufficient to induce macrophage pro-inflammatory activation

**Fig 3:**
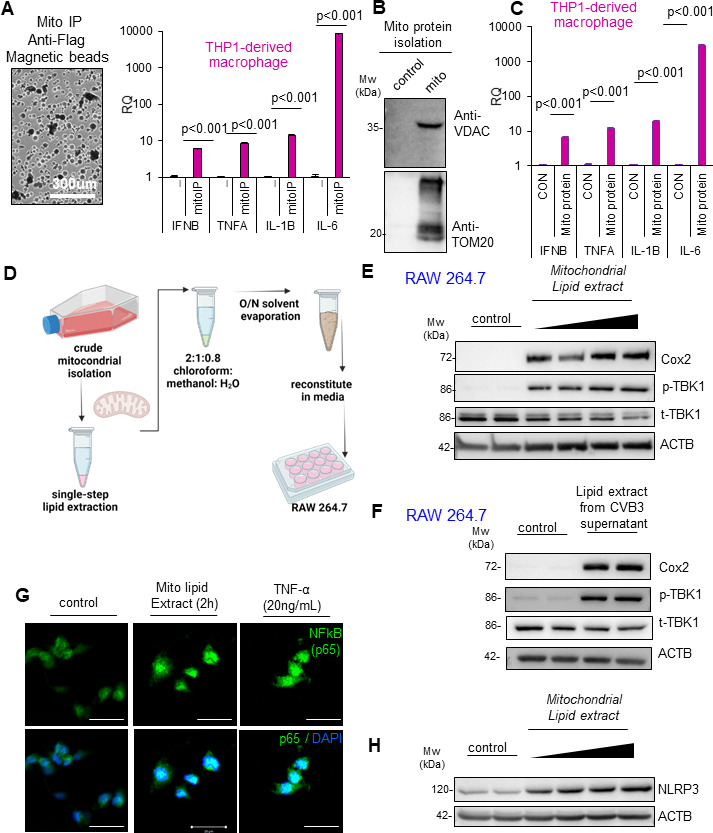
Incubation with mitochondrial proteins and lipids induces macrophage pro-inflammatory activation. (**A**) Bright field image of mitochondria isolated using mito-IP protocol (left). THP1-derived macrophages were treated with mitochondria purified from mito-IP diluted in culture medium (right). Total RNA was purified from cells and subjected to qPCR analysis for inflammatory gene markers. (**B**) Proteins were extracted from mito-IP preparations and subjected to Western blot analysis for mitochondrial markers VDAC and TOM20. Statistical analysis between control and mito-IP groups was performed using an unpaired Student *t*-test (mean ± SD, *n* = 3). (**C**) Purified mitochondrial protein fractions (**B**) were diluted in culture medium and used to treat THP1-derived macrophages for 4 h. RNA was purified from cells following treatment and subjected to qPCR measurement for inflammatory gene expression. Statistical analysis between control and mito protein groups was performed using an unpaired Student *t*-test (mean ± SD, *n* = 3). (**D**) Schematic illustration of lipid isolation protocol from purified crude mitochondria extracts. (**E**) RAW264.7 macrophage cells were treated with mitochondrial lipid extracts from panel **D** diluted in culture medium for 2 h and then subjected to Western blot analysis for macrophage activation marker COX2, p-TBK1, and t-TBK1. ACTB was used as a loading control. (**F**) Lipids were extracted from CVB3 supernatant preps as in panel **D** and diluted in culture medium, followed by 2 h incubation with RAW264.7 macrophage cells. Cells were subjected to Western blot analysis for macrophage activation marker COX2, p-TBK1, and t-TBK1. (**G**) RAW264.7 cells were incubated with mitochondrial lipid extracts as in panel** E**and then subjected to confocal microscopy analysis for NFkB (P65) nuclear localization. Cells treated with TNF-α (20 ng/mL, 60 min) were included as a positive control for NFkB nuclear translocation. Scale bar = 20 µm (**H**) RAW264.7 macrophages from panel** E** were assessed by Western blot analysis for protein expression of NFkB downstream inflammatory gene NLRP3. ACTB served as a loading control.

As assays of CVB3 viral protein were negative in infected macrophage, we focused on the inflammatory effects of CVB3 exposure. Indeed, RAW264.7 macrophages demonstrated robust sensitivity and inflammatory responsiveness to CVB3 treatment. Given this response and the previous exclusion of mtDNA as a strong driver of macrophage activation, we sought to test the role of other mitochondrial components as a potential mechanism for CVB3-induced macrophage activation. In addition to pathogen-associated molecular patterns such as viral proteins or genomes, damage-associated molecular patterns (DAMPs) originating from infected host cells can also robustly initiate pathological inflammation (14). Considering the high concentration of mitochondria in cardiac tissue, their involvement in anti-viral defense, the observed changes to mitochondrial networks after infection, and the release of mitochondrial components during infection, we investigated whether mitochondria might trigger pro-inflammatory signaling in THP-1 derived macrophage cells. Interestingly, THP1-derived macrophages incubated with culture medium containing mitochondrial immunoprecipitation (mito-IP) isolated mitochondria (as outlined in Materials and Methods) demonstrated significant induction of pro-inflammatory cytokines *IFNB*, *TNFA*, *IL-1B*, and *IL-6* (Fig. 3A). Moreover, mitochondrial protein isolated from mito-IP preparations was equally potent in stimulating pro-inflammatory signaling in THP1-derived macrophages (Fig. 3B and C). Protein isolates were subjected to immunoblotting and demonstrated positive staining for the mitochondria localized proteins VDAC and TOM20.

In addition to total mitochondria and mitochondrial proteins, we sought to test the role of mitochondrial lipid extracts in pro-inflammatory signaling. Mitochondrial extracts were subjected to single-step lipid extraction protocol, which was reconstituted in culture media. Prior to lipid extraction, mitochondrial isolates were assessed for mitochondrial protein markers VDAC and TOM20 (Fig. S3). Subsequently, RAW264.7 macrophages were incubated with media containing mitochondrial lipid extracts (Fig. 3D). Western blot analysis of cell lysates revealed robust expression of macrophage activation marker COX2 upon treatment with increasing dose of mitochondrial lipid extracts as well as activation of the innate immune kinase TBK1 (Fig. 3E). Remarkably, treatment of RAW264.7 macrophages with lipid extracts alone from CVB3 supernatants was sufficient to induce macrophage and pro-inflammatory activation (Fig. 3F). Mitochondrial lipid extracts also demonstrated the capacity to activate NFκB (p65) transcription factor nuclear localization in treated RAW264.7 cells similar to TNFα cytokine that served as a positive control (Fig. 3G). Lastly, RAW264.7 cells treated with mitochondrial lipid extracts robustly activated downstream NFκB target gene NLRP3 in a dose-dependent manner (Fig. 3H). Altogether, this evidence suggests that mitochondrial components, including mitochondrial proteins and lipids, can potently activate pro-inflammatory signaling in macrophages.

### CVB3 infection is associated with pathological complement activation

**Fig 4:**
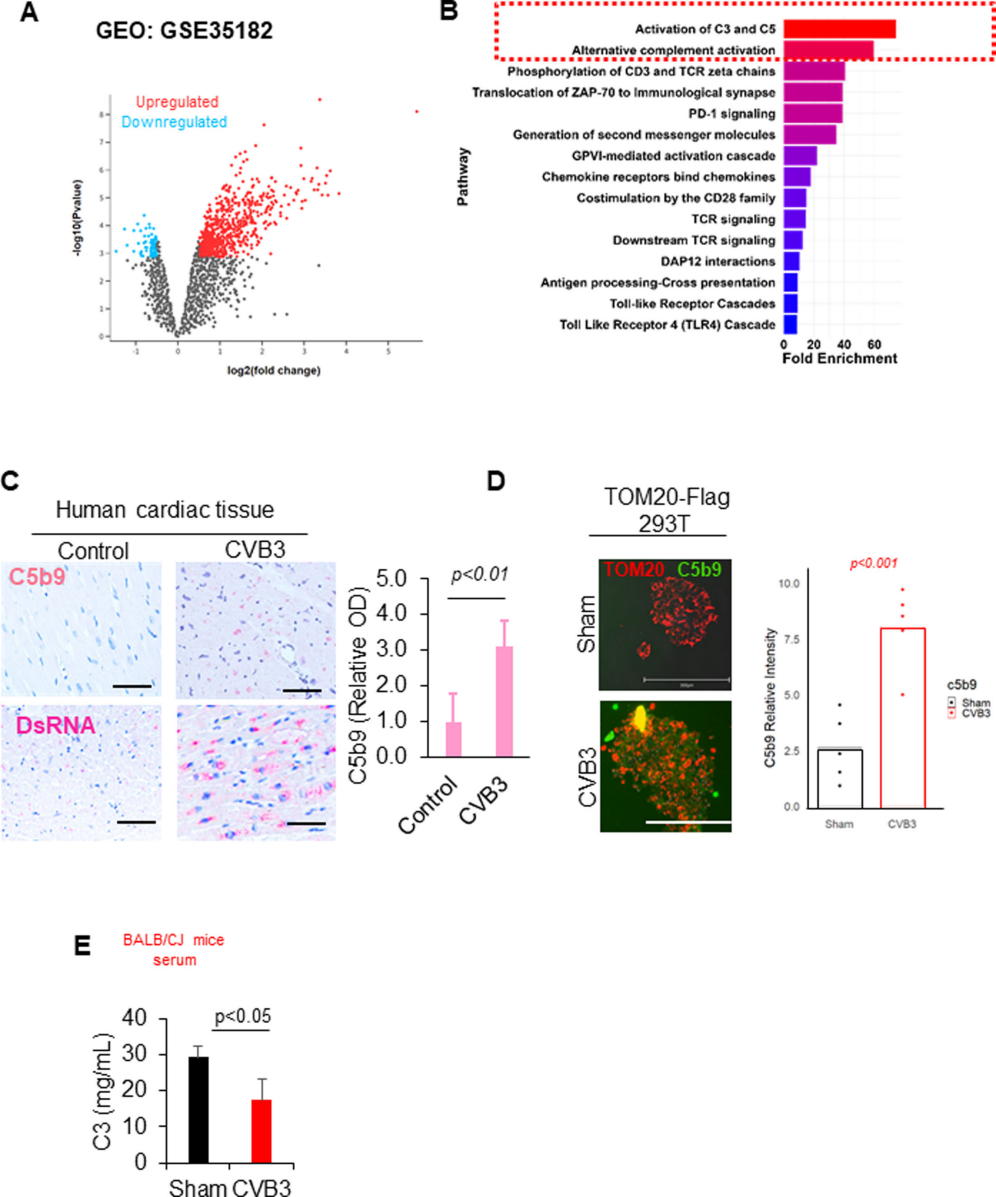
CVB3 infection is associated with pathological complement activation. (**A**) Volcano plot demonstrating differential gene expression between sham- and CVB3-infected male BALB/c mice 6–8 weeks old at 10 days post-infection, with red points representing significantly upregulated genes and blue points indicating significantly downregulated genes (*n* = 3 mice per group). (**B**) Analysis of differentially upregulated genes in panel** A** was plotted using R studio and presented in rank order of highest enrichment using Panther’s annotated Reactome data set. (**C**) CVB3-positive human myocarditis tissues exhibit elevated levels of membrane attack complex C5b9. Tissues were stained with double-stranded RNA (dsRNA) as a marker of viral RNA replication and confirmed with *in situ* hybridization CVB3-specific probes (see reference [16]). Scale bar = 50 µm. Results are representative of three biologically independent samples for each group. C5b9 optical density was quantified in the right panel and presented as mean ± SD, *n* = 3. (**D**) TOM20-expressing HEK293 cells were sham or CVB3-infected for 8 h at an MOI of 100 and then analyzed by immunofluorescence staining with anti-TOM20 (red) and anti-C5b9 (green) antibodies. Relative C5b9 intensity was assessed between sham and CVB3-infected cells and quantified in the right panel (mean ± SD, *n* ≥ 50 cells per condition). Statistical analysis between sham and CVB3 groups was performed using an unpaired Student *t*-test. Scale bar = 300 µm (**E**) Complement protein C3 was measured through C3-specific enzyme-linked immunosorbent assay in the serum of sham or CVB3-infected BALB/c mice. Statistical analysis between sham and CVB3 groups was performed using an unpaired Student *t*-test (mean ± SD, *n* = 3). OD, optical density.

Mitochondrial components, originating from ancient protobacteria, retain molecular patterns that are recognized by the immune system’s evolutionarily conserved anti-bacterial defenses, including the complement pathway (7). Before investigating the mitochondria-complement cross talk, we first inquired whether complement activation occurs during CVB3 infection. To better understand the role of complement in CVB3 infection, we sought to evaluate whether complement activation was present in published studies of viral myocarditis. To this end, we explored the public genomics data repository, Gene Expression Omnibus (GEO), and identified a previous gene expression study on CVB3-induced viral myocarditis (GSE35182) (15). Given that the risks and pathogenesis of viral myocarditis are higher in males, we examined differential gene expression in control PBS-treated and 10-day CVB3-infected male BALB/c mice (Fig. 4A). Pathway analysis revealed significant enrichment and overrepresentation of genes associated with the activation of complement C3 and C5 as well as alternative complement activation (Fig. 4B). Leveraging the unique resources of the McManus Cardiovascular Biobank, we identified human cardiac tissues from clinically confirmed viral myocarditis samples. CVB3 positivity was confirmed through *in situ* hybridization as previously described (16), although it cannot be excluded that the probe used could cross react with other species of *Enterovirus B*. Compared to control cardiac tissue from normal donors, CVB3-positive samples demonstrated elevated staining for dsRNA, a marker of viral genome replication within cytosolic compartments of cardiomyocytes (Fig. 4C). To determine the involvement of the complement pathway in the pathogenesis of viral myocarditis, we conducted immunohistochemical staining on these human samples using an antibody against C5b-9, a marker of the membrane attack complex in the complement system (17). We demonstrated elevated staining for C5b-9 in virus-infected hearts compared to control hearts (Fig. 4C). Consistent with this, CVB3-infected TOM20-stable cells also showed elevated immunostaining of the C5b-9 marker (Fig. 4D). Similarly, CVB3-infected mice had reduced fluid phase complement protein C3 in the serum compared to sham-infected mice (Fig. 4E). Together, this evidence suggests that CVB3 infection is associated with pathological activation of the terminal complement pathway.

### Mitochondria-mediated activation of complement exacerbates inflammation

**Fig 5:**
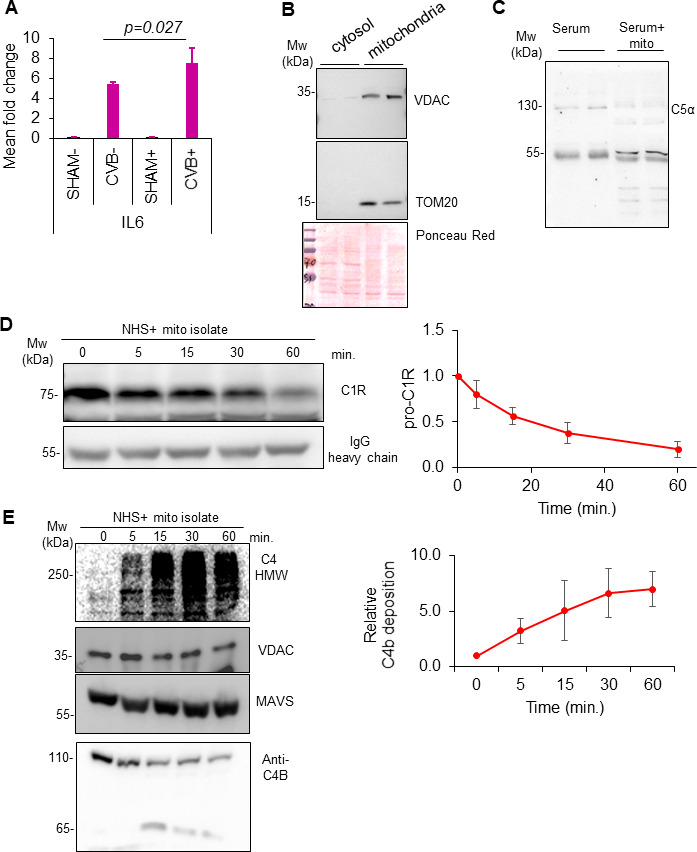
Mitochondria-mediated activation of complement exacerbates inflammation. (**A**) Serum complement factors enhance CVB3-induced pro-inflammatory macrophage activation. RAW264.7 macrophages were either sham or CVB3-infected in the presence (+) or absence (−) of 10% normal human sera (NHS). RNA was extracted and subjected to qPCR analysis for IL-6 and presented as mean fold change relative to −NHS/sham (group 1). Statistical analysis between groups was performed using analysis of variance with Tukey post hoc test. (**B**) Purified mitochondria from stable TOM20-HEK293 cells were subjected to Western blot analysis for mitochondrial proteins VDAC and TOM20. Ponceau red staining was used as a total protein loading control. (**C**) NHS was incubated with or without purified mitochondria for 1 h at 37°C, and the mixture was subjected to Western blot analysis for complement protein C5. (**D and E**) Purified mitochondria were subjected to *in vitro* complement time-course assay in the presence of anti-TOM20 antibody. Lysates were subjected to Western blot analysis for complement factors component 1R (C1R) (**D**) and component 4 (C4) (**E**). Complement activation was assessed and quantified in the right panels. These results are representative of three independent biological experiments (mean ± SD, *n* = 3).

We next sought to link virus-induced mitochondrial damage and release to pathological complement activation observed in cases of viral myocarditis. Given that mitochondria are remnants of an ancient bacterial origin and the complement system has evolutionary ties to innate anti-bacterial defense (8), we proceeded to test the direct interactions between mitochondria and the complement system. First, we evaluated whether complement factors could exacerbate macrophage-mediated inflammation. Indeed, RAW264.7 cells cultured with complement-containing serum showed a significant elevation in the gene expression of inflammatory cytokine IL-6 compared to serum-free cells (Fig. 5A). To directly assess the role of mitochondria in complement activation, mitochondria were isolated using mito-IP as above and verified for mitochondrial proteins TOM20 and VDAC (Fig. 5B). When mito-IP was incubated *in vitro* with normal human serum, activated C5 processing was observed (Fig. 5C). Moreover, time-course *in vitro* assays with purified mitochondria and normal human serum exhibited a time-dependent increase in complement component 1R proteolytic processing and high-molecular-weight complement component 4 (C4) accumulation and processing, suggesting that virus-induced mitochondrial release can directly activate complement pathways (Fig. 5D and E).

### Complement protective factors are downregulated following CVB3 infection

**Fig 6:**
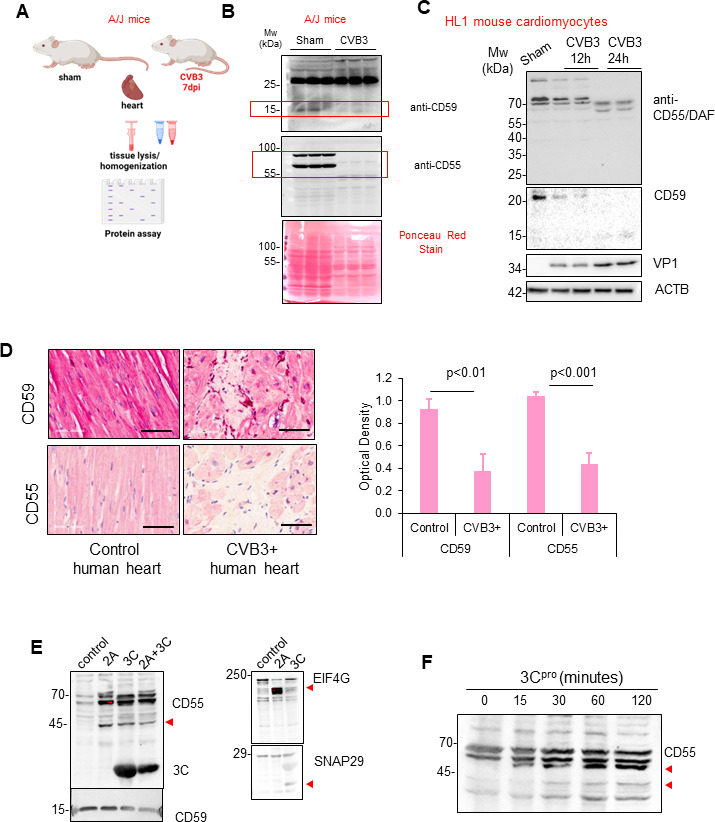
Complement protective factors are downregulated following CVB3 infection. (**A**) Schematic illustration of cardiac protein expression analysis in control and 7 day CVB3-infected A/J male mice (*n* = 3 per group). (**B**) Protein expression of complement protective factors CD59 and CD55 was assessed by Western blot analysis from cardiac tissue of sham or CVB3-infected A/J mice (**A**). Ponceau red stain of membrane was used to assess total protein. (**C**) HL1 cardiomyocytes were sham or CVB3-infected (MOI = 100) for 12 or 24 h. Proteins were harvested from cells and subjected to Western blot analysis for complement protective factors CD55 and CD59. VP1 and ACTB were used as viral replication marker and loading control, respectively. (**D**) Control and CVB3-infected human heart tissues were subjected to immunohistochemistry to detect the complement protective factors CD59 and CD55. Scale bar = 60 µm. CD59 and CD55 optical density was quantified in the right panel and presented as mean ± SD, *n* = 3. (**E**) *In vitro* cleavage assay was performed on HeLa cell lysates using purified CVB3 viral proteinase 2A (300 ng), 3C (100 ng), or 2A + 3C (left). Previously reported substrates EIF4G and SNAP29 were used as positive controls for 2A and 3C, respectively (right). Red arrows depict cleavage fragments. (**F**) Time-course *in vitro* cleavage assay was performed using HeLa cell lysates treated with purified viral proteinase 3C (100 ng). Lysates were subjected to Western blot analysis for CD55. Red arrowheads depict cleavage fragments.

To further elucidate the mechanism behind CVB3-induced complement activation, we examined the effects of CVB3 infection on complement protective factors. CD59, also known as protectin, and CD55, known as decay-accelerating factor (DAF), serve as crucial regulators that safeguard cells against self-inflicted damage by inhibiting complement activation and thus preventing autoimmune attacks (18). Using the established viral myocarditis model, male A/J mice were infected with CVB3, and cardiac tissue was harvested at 7 days post-infection, coincident with the severe inflammatory stage of this disease (Fig. 6A). Western blot analysis of cardiac tissue from sham and CVB3-infected mice revealed significant downregulation of complement protective factors CD59 and CD55 (Fig. 6B). Total protein levels evaluated by ponceau stain of control and infected murine cardiac tissue revealed global protein downregulation in infected samples. Consistent with this observation, HL1 cardiomyocytes infected with CVB3 demonstrated a time-dependent downregulation of CD55 and CD59 (Fig. 6C). Of interest, reduction in both full-length CD55 and CD59 proteins was accompanied by the emergence of lower- molecular-weight fragments, suggestive of proteolytic processing. Downregulation of complement protective factors was also observed in virus-positive human cardiac tissue (Fig. 6D). Mechanistically, we sought to test the role of viral proteinases in CD55 downregulation. We focused our efforts on CD55 as its molecular size allowed for easier detection of cleavage fragments compared to the much smaller CD59 protein. To that end, we performed a series of *in vitro* cleavage assays utilizing recombinant purified viral proteinases 2A and 3C. Interestingly, both viral proteinase 2A and 3C were able to cleave CD55 and generate at least two proteolytic fragments within the approximate range of 45–55 kDa (Fig. 6E and F). Interestingly, treatment of cells with viral proteinase 3C alone was not sufficient to significantly alter cell viability (Fig. S4), suggesting that additional viral and/or host factors may be involved in viral cell injury. Together, our data suggest that CVB3 downregulates complement protective factors CD55 and CD59, at least in part through viral proteinase-mediated cleavage, thereby contributing to complement activation.

## DISCUSSION

Mitochondria are theorized to have originated from ancient proteobacteria that established a symbiotic relationship with eukaryotic cells (19, 20). Beyond their physiological role in energy production, mitochondrial dysfunction is increasingly being recognized as a driver of disease pathophysiology particularly in mitochondria-enriched tissues such as the central nervous system and the heart (11). While the diverse mechanisms of mitochondrial dysfunction in various diseases are actively being investigated, the role of viral infection in the development of myocarditis is becoming increasingly apparent. Viral myocarditis is an inflammatory disease that affects about 100 individuals per 100,000 worldwide (21). While most instances of myocarditis can resolve spontaneously, up to 30% of patients can progress to viral heart failure (22). The differential responses in myocarditis cases may be attributed to variations in individual immune responses, viral virulence, and genetic predispositions affecting the heart’s ability to heal and manage inflammation.

Recognizing the critical role of mitochondria—the most abundant organelles in heart tissue that is central to cell death, inflammation, and energy demands—we focused on mitochondrial dysfunction, a well-known hallmark of heart failure (10). To gain further insight into how mitochondrial dysfunction influences heart disease, we strategically utilized RNA expression data from human heart tissue in the GTEX database (23). This allowed us to segment cardiac tissue into categories of low and high mitochondrial abundance, serving as a proxy measurement to identify mitochondrial dysfunction potentially linked to inflammatory activation.

Our analysis revealed that cardiac tissues with low mitochondrial abundance not only showed increased expression of pro-inflammatory genes but also exhibited elevated complement activation. This finding supports a potential connection between diminished mitochondrial integrity and the observed enhancement of inflammatory and complement responses in cardiac tissues. Consequently, we used these insights to further explore the role of mitochondria in the pathogenesis of viral myocarditis.

Building on this foundation, we turned our attention to the cellular mediators of inflammation, particularly focusing on the role of macrophages during CVB3 infection. Despite not being permissive to CVB3 replication (24), our study demonstrated that macrophages were highly responsive to CVB3 infection and virus-associated DAMPs, including components released from mitochondria. These observations are consistent with a previous finding that mitochondria co-transmits with CVB3 during viral propagation (25). *In vitro* experiments further elucidated that CVB3-induced mitochondrial injury leads to the release of mtDNA into the cytosol. Interestingly, it was recently shown that viral infection of RAW264.7 macrophages did not activate the cGAS-STING pathway (24), which is typically involved in cytosolic DNA sensing (26–28). Similarly, a recent study demonstrated that CVB3 infection can bypass cGAS-STING activation in cells that are directly infected with CVB3 (28). In contrast, Qin et al. reported that mtDNA can trigger STING-dependent inflammation in a viral myocarditis model, partly through macrophage upregulation of STING, although the detailed mechanism was not clearly defined (29). On the other hand, our study showed the inflammatory response in macrophages was predominantly driven by the activation of the NFκB pathway. This pathway’s activation was notably independent of viral replication and traditional DNA sensing mechanisms (24), suggesting a novel inflammatory pathway triggered by mitochondrial dysfunction and viral components interacting with macrophages. Given the differential permissiveness of viral replication in cardiomyocytes vs macrophages, a likely model may involve an initial viral-mediated mitochondrial injury in cardiomyocytes that acts as a source of DAMPs to further trigger macrophages during infection. These findings point to the importance of host-derived DAMPs (e.g., mitochondrial lipids) in the pathological inflammatory process and highlight the need for future studies to explore multipronged therapeutic strategies that address the consequential byproducts of viral-mediated injury.

**Fig 7:**
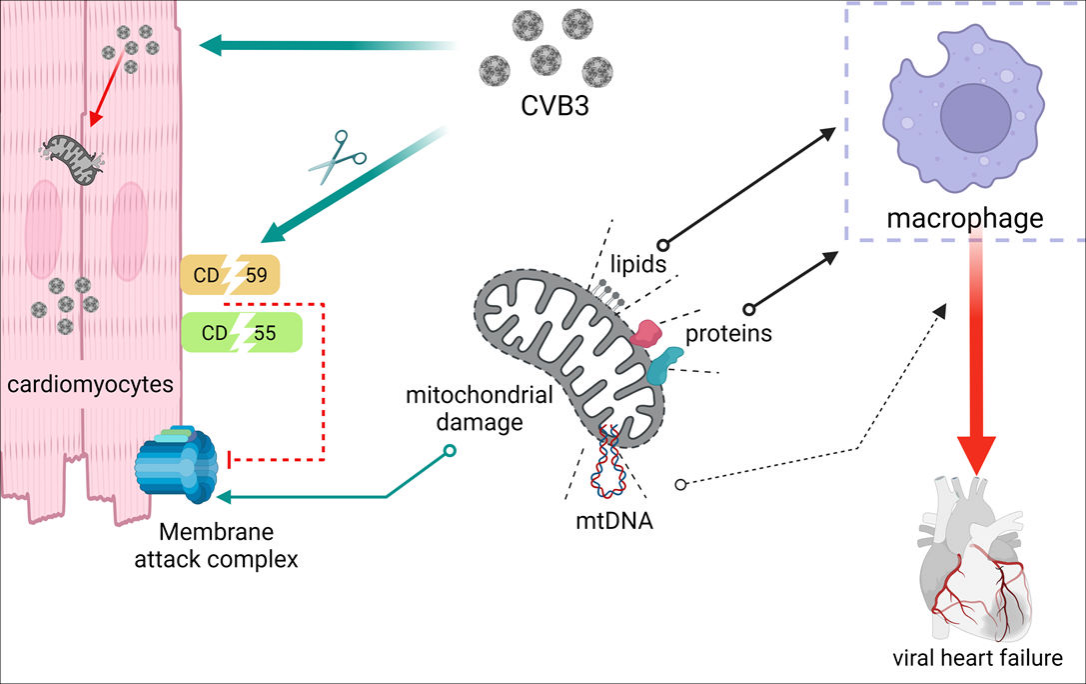
Schematic illustration of the novel mechanism that can act as a potential contributor to CVB3 pathogenesis through mitochondrial injury-mediated autoimmunity.

The role of mitochondrial components in activating inflammatory pathways was further examined in our study, which demonstrated that mitochondrial proteins and lipids could independently stimulate pro-inflammatory pathways in macrophages (Fig. 7). This underscores the potent inflammatory potential of mitochondrial components, particularly when released during viral infection. To further understand the role of mitochondria in immune modulation, we investigated their ability to activate the complement system. Previous studies have suggested that mitochondrial components, such as the inner membrane lipid cardiolipin, may become exposed during mitochondrial injury and activate the complement pathway (30). In the current study, we demonstrate that mitochondrial isolates can directly activate the classical complement pathway in the presence of anti-mitochondrial antibodies supplemented with the isolates. While rare instances of cardiac injury have been associated with anti-cardiolipin antibodies in patients, whether this mechanism is involved in viral heart failure cases remains to be determined (31).

Additionally, we observed significant downregulation of complement protective factors such as CD59/protectin and CD55/DAF in CVB3-infected cardiac tissues. Although enteroviruses have been shown to shut off global protein translation through targeting of translation initiation factors (32), our study revealed additional mechanism of direct protein targeting. The reduction of CD55 and CD59 appears to be facilitated at least in part by the activity of CVB3-specific proteinases, which cleave these protective proteins, potentially exacerbating the inflammatory response and complement activation. Another possible mechanism for the downregulation of these proteins could involve viral receptor modulation. Indeed, other studies have shown that viruses can downregulate their entry receptors post-infection (e.g., ACE2 and CD4 downregulation following severe acute respiratory syndrome coronavirus 2 and HIV infections, respectively) (33, 34). Given that CVB3 uses CD55/DAF as a co-entry receptor, this may be an additional mechanism through which CVB3 downregulates complement protective proteins. Interestingly, Yanagawa and colleagues previously demonstrated that recombinant soluble CD55/DAF-Fc fusion protein indeed can provide cardioprotection following CVB3 infection (35). Beyond innate immunity, complement factors may intersect with adaptive immunity during myocarditis. For example, Kaya et al. demonstrated that complement is crucial for inducing experimental autoimmune myocarditis through complement receptor type 1 (CR1) and complement receptor type 2 (CR2). They identified a subset of T cells expressing CR1 and CR2 and proposed these receptors may be involved in B- and T-cell activation, T-cell proliferation, and cytokine production. Together, these lines of evidence suggest a mechanism by which viral-mediated activation of complement can modulate autoimmune disease (36).

Collectively, findings from this study illustrate a complex interplay between viral infection, mitochondrial perturbation, and complement system involvement in the development of viral myocarditis. This intricate relationship may highlight the critical role of the complement system not only in viral myocarditis but also in broader cardiac conditions. For instance, a study by Shahini et al. revealed complement dysregulation in patients with chronic heart failure, noting particularly that low levels of complement factor H, a protective factor, were associated with worse outcomes (37). Furthermore, another investigation found significant elevations in circulating C5b-9, the terminal complement complex, in patients with symptomatic heart failure and linked high levels of C5b-9 with adverse events (38). Adding to the complexity of heart failure’s interaction with the complement system, cases of cardiac dysfunction during sepsis, referred to as “septic cardiomyopathy,” have been attributed to the intensive activation of the complement system, with specific findings indicating that production of the anaphylatoxin C5a contributed to cardiac dysfunction, which could be ameliorated by anti-C5a antibodies (39).

Given that pathological complement activation is evident in various cases of heart failure, numerous studies have pursued complement blockade as a strategy to protect the heart from complement-mediated injury. Intravenous immunoglobulins (IVIG) in high dosages, for example, are increasingly being used to treat a range of inflammatory disorders and have demonstrated efficacy in improving left ventricular ejection fraction in congestive heart failure (40). IVIG can reduce complement-mediated tissue damage through mechanisms like binding activated C3 and C4, diverting C1q from the target to the fluid phase, and enhancing C3b inactivation. IVIG improves cardiac function in congestive heart failure patients, but excessive activation of complement in the fluid phase may counteract these benefits by producing inflammatory substances (40).

In response to the limitations of broad immunomodulators like IVIG, more targeted complement therapies have emerged, such as the monoclonal antibody eculizumab, which targets C5 and has been proven effective in conditions like atypical hemolytic uremic syndrome and paroxysmal nocturnal hemoglobinuria (41, 42). Indeed, several studies have shown that blocking the complement system with anti-C5 antibodies can enhance outcomes in heart failure, particularly in cases of antibody-mediated rejection following heart transplantation (43–45). Whether such therapy can improve viral myocarditis outcomes and the optimal timing of treatment initiation in the clinic needs further investigation. Interestingly, a recent study has implicated genetic variations in complement regulatory proteins, such as complement factor H, in the pathogenesis of coronavirus disease 2019 (46). These insights into how viral infections interact with innate immune pathways, such as the complement system, open promising research avenues for developing more effective therapy.

## MATERIALS AND METHODS

### Animal infections

Male A/J (#000646, *n*  = 3 sham, *n* = 3 CVB3) were acquired from Jackson Laboratories (Bar Harbor, ME, USA). Mice at age 4–6 weeks were infected with sham (PBS) or CVB3 (10^4^ PFU) through intraperitoneal administration as previously described (47). Mice were administered inhalant anesthetic isoflurane prior to CO_2_ euthanasia according to the local animal care guidelines. Mice were sacrificed on day 7 after viral infection in a CO_2_ chamber, and hearts were harvested. Female BALB/c mice (000651, Jackson Laboratories, *n* = 4 sham, *n* = 3 CVB3), aged between 6 and 8 weeks, were used for C3 measurement in sera (48).

### Cell culture and viral infection

HL-1 cell line (mouse atrial cardiomyocyte) was a kind gift from W. Claycomb (Louisiana State University Medical Center, New Orleans, LA, USA) and cultured in Claycomb medium (Millipore Sigma, cat #51,800C) supplemented with 4 mM glutamine, 100 µM norepinephrine, and 10% fetal bovine serum (FBS). THP-1 human monocyte cell line was a kind gift from G. Francis (Centre for Heart Lung Innovation, University of British Columbia) and cultured in RPMI-1640 medium supplemented with 10% FBS. For differentiation into macrophages, THP-1 suspension cells were incubated overnight with 500 nM of phorbol 12-myristate-13-acetate. RAW 264.7 mouse macrophage cells, HeLa and HEK293 cells (American Type Culture Collection, TIB-71CCL-2 and CRL-1573), were cultured in Dulbecco’s Modified Eagle’s Medium (DMEM) supplemented with 10% FBS and a penicillin-streptomycin cocktail (100 µg/mL). For CVB3 infection, cells were either sham infected with PBS (Sigma, D8537) or inoculated with CVB3 (Kandolf strain; generously provided by Reinhard Kandolf, University of Tubingen, Germany) at different MOIs as specified in the figure legends.

### Immunohistochemistry (IHC)

Human cardiac tissue samples were obtained from the Bruce McManus Cardiovascular Biobank. Normal cardiac tissues used as controls were obtained from autopsy, whereas viral positive cardiac samples were obtained from diseased transplant hearts having undergone clinical diagnosis and *in situ* hybridization (RNAscope)-based confirmation for virus-induced heart failure (49). IHC was performed on formalin-fixed paraffin-embedded human cardiac sections using a Bond polymer refined red detection kit (cat #DS9390; Leica Biosystems Inc., Buffalo Grove, IL, USA) on the Leica Bond Autostainer according to the manufacturer’s guidelines. Stained slides were dried overnight, coverslipped, and scanned using the Aperio scanning system (Leica Biosystems Inc.) for further analysis. Primary antibodies used for staining include CD59 (Abcam, ab9183), CD55 (Abcam, ab231061), TOM20 (Abclonal, A19403), dsRNA J2 ms mAb (Scicon, 10010500), and cGAS (Abclonal, A8335).

### Immunofluorescence and confocal microscopy

HL1, HEK293T-3×Flag-TOM20 stable cells, and RAW 264.7 cells were cultured in eight-well chamber slides (Labtek, 155411) for 24 h prior to treatments. Sham and infected cells were fixed at 37°C for 30 min in 4% paraformaldehyde/100 mM sucrose fixative. Formaldehyde was quenched with three rinses of 0.1 M glycine/PBS, and cells were permeabilized with 0.1% Trition X-100 (Sigma, T8787) for 3 min, followed by 1 h blocking in 3% bovine serum albumin (BSA) (Sigma A7030)/PBS. Cells were incubated overnight with primary antibody diluted in 3% BSA/PBS following the manufacturer’s dilution recommendation. Upon rinsing primary antibody with PBS (three washes of 5 min each), cells were incubated for an additional 1 h in Alexa Fluor conjugated secondary antibody followed by subsequent rinses as above. Cells were mounted with Fluoroshield and 4, 6-diamidino-2-phenylindole (Sigma-Aldrich, F6057). Immunofluorescence images were captured on the EVOS M5000, and confocal images were captured with the Zeiss LSM 880 Inverted Confocal Microscopy using a ×63 objective lens.

### GEO and GTEX analysis

Gene expression data of control and acutely CVB3-infected male mice (BALB/c mice 6–8 weeks old, 10 days post-infection) was extracted from Coronado et al. (15) (GEO accession ID: GSE35182). GEO2R analysis tool was used to assess differentially expressed genes. Top differentially expressed genes (adjusted *P* value of <0.001) were analyzed by Gene Ontology Enrichment Analysis: PANTHER Overrepresentation Test and the annotation data set Reactome pathways (Reactome version 85). A test type of Fisher’s exact with a false discovery rate correction (*P* < 0.05) was used. Enriched reactome pathways were plotted using ggplot2 package in R studio (2022.12.0 Build 353).

For human left ventricle gene expression analysis, the GTEX database was used. Raw data from the gene reads were processed in R Studio (2022.12.0 Build 353) and Microsoft Excel (2019). Samples were segmented into low-mitochondria and high-mitochondria groups based on mtCO1, and the top and bottom 27 samples were used for further downstream analysis. Differential gene expression of select inflammatory gene targets was visualized using the publicly available web server Heatmapper (50).

### Western blot analysis

Western blot analysis was performed as previously described (51). Briefly, cells were lysed on ice with modified optimal salt lysis buffer (10 mM HEPES, pH 7.4, 50 mM sodium pyrophosphate, 50 mM NaF, 50 mM NaCl, 5 mM EDTA, 5 mM EGTA, 100 µM Na3VO4, and 0.1% Triton X-100). Lysates were spun at 13,000 rpm to pellet debris, and approximately 30 µg of protein was loaded with subjected to SDS-PAGE. Western blotting was conducted using the following primary antibodies: Cox2 (D5H5) (Cell Signaling Technology, #13314), p-TBK1 (Cell Signaling Technology, #5483), TBK1 (Cell Signaling Technology, #3504), STING (Cell Signaling Technology, #13647), p-STING (Cell Signaling Technology, #19781), VP1 (Mediagnost, Cox mAb 31A2), NLRP3 (Adipogen, #AG-20B-0014-C100), VDAC (Abclonal, #A19707), TOM20 (Abclonal, #19403), ACTB (Sigma-Aldrich, A5316), FLAG (Sigma, F1804), CD59 (Abclonal, #A4090), CD55 (Abcam, #ab231061), C4B (Abcam, #ab181241), LC3B (Novus Biologicals, NB100-2220), CAR (Cell Signaling Technology, #16984), and MAVS (Cell Signaling Technology, #24930).

### Mito-IP

Mitochondria was isolated using an immunoprecipitation protocol as outlined below. In brief, HEK293 cells stably expressing 3× Flag TOM20 were lysed using a standard Dounce homogenization (60 strokes on ice). Cell homogenates were spun at 700 × *g* for 10 min at 4°C to pellet cell debris and nuclei. Supernatants containing cytosolic and mitochondrial fractions were incubated overnight in 4°C with Anti-FLAG M2 Magnetic Beads (Sigma-Aldrich, M8823) according to the manufacturer’s instructions. After three washes in Tris-buffered saline wash buffer, the bound proteins/organelles were eluted with 2 × SDS sample buffer for Western blot analysis or subjected to mechanical separation through extended vigorous vortexing (5 min) in PBS. Dissociated eluates were validated for mitochondrial content through protein and mtDNA analysis.

### Mitochondrial protein and lipid extraction

Following mito-IP, eluates in PBS were subjected to protein harvesting as previously described (52). Briefly, DynaMag-Spin Magnet separator (Invitrogen, #12320D) was used to filter FLAG M2 magnetic beads from the mitochondrial eluates in PBS. Mitochondrial protein was precipitated by an equal volume of methanol and 0.25 vol of chloroform as described previously (53). Briefly, the supernatant, methanol, and chloroform mixture was vortexed and centrifuged for 10 min at 20,000 × *g* at 4°C. Upon removing the upper phase, an additional volume (500 µL) of methanol was added to the interphase and subjected to 10 min spin at 20,000 × *g* at 4°C. Protein pellet was dried at 55°C and resuspended in PBS for downstream use. Mitochondrial lipid was extracted using a single-step lipid extraction protocol as previously described (54). Briefly, mitochondrial eluates were mixed in a 2.0:1.0:0.8 vol mixture of chloroform, methanol, and 0.73% NaCl-H_2_O solution, respectively. Following overnight solvent evaporation, lipid pellets were reconstituted in culture medium for subsequent experiments.

### Hypotonic lysis

Sham or CVB3-infected HL1 cells were subjected to hypotonic lysis as previously described (55). Briefly, cells were incubated with hypotonic lysis buffer (20 mM HEPES, pH 7.4, 10 mM KCl with phosphatase, and protease inhibitors) for 20 min on ice. Cells were further homogenized by passing then through a 25-gauge needle for a total of 15 times, followed by a brief centrifugation to remove nuclear and cell debris. Supernatants were mixed with an equal volume of 2× DMEM culture media and used for downstream treatment of RAW264.7 cells.

### mtDNA measurement

To assess mtDNA amounts, purified mitochondria were subjected to DNA extraction using a conventional DNA isolation protocol as previously described (56) with the Monarch Plasmid Miniprep Kit (New England Biolabs, #T1010L). Extracted mtDNA was assessed for purity using NanoDrop 8000 Spectrophotometer. Relative mtDNA quantity was compared between control and experimental conditions using quantitative PCR with the specific mtDNA primer targeting the *mtCO2* gene (Table 1).

**TABLE 1:** Primer sequences for RT-qPCR used in this study

	Forward	Reverse
Ifnb1 (mouse)	GCCTTTGCCATCCAAGAGATGC	ACACTGTCTGCTGGTGGAGTTC
IFNB (human)	CAACTTGCTTGGATTCCTACAAAG	TATTCAAGCCTCCCATTCAATTG
Il6 (mouse)	ACAACCACGGCCTTCCCTAC	TCTCATTTCCACGATTTCCCAG
IL6 (human)	ACTCACCTCTTCAGAACGAATTG	CCATCTTTGGAAGGTTCAGGTTG
Il1b (mouse)	GCAACTGTTCCTGAACTCAACT	ATCTTTTGGGGTCCGTCAACT
IL-1B (human)	CCACAGACCTTCCAGGAGAATG	GTGCAGTTCAGTGATCGTACAGG
Cxcl10 (mouse)	GCTGGGATTCACCTCAAGAA	CTTGGGGACACCTTTTAGCA
Ccl5 (mouse)	GCTGCTTTGCCTACCTCTCC	TCGAGTGACAAACACGACTGC
TNFA (human)	CCTCTCTCTAATCAGCCCTCTG	GAGGACCTGGGAGTAGATGAG
MTCO2 (human)	AATCGAGTAGTACTCCCGATTG	TTCTAGGACGATGGGCATGAAA

### mtDNA depletion assay

HEK293T-3×Flag TOM20 stable cells were subjected to control treatment or mtDNA depletion using ddC as previously described (57). Briefly, cells were incubated with control DMEM medium or medium containing 50 µg/mL of ddC for 144 h with a medium change taking place at the 72 h timepoint. Mitochondria and mtDNA were respectively isolated using mito-IP and DNA isolation protocols as described above. Isolated mtDNA was assessed via acrylamide gel electrophoresis following *HindIII* restriction enzyme digestion.

### Complement 3 enzyme-linked immunosorbent assay measurement

C3 protein levels were quantified from serum of control and CVB3-infected BALB/c mice using a commercially available mouse C3 ELISA Kit (Abcam, #AB263884) according to the manufacturer’s protocol.

### Complement activation assay

Complement serum from mouse (Sigma, cat #S3269-5mL) was reconstituted in deionized water and aliquoted/stored in −80°C. Normal human sera (NHS) was purchased from Comptech (#NHS). Mitochondria were extracted using the mitochondrial isolation kit for cultured cells (Thermo Fisher, cat #89874) according to the manufacturer’s protocol. Briefly, two T75 flasks (~2 × 10^7^ cells) of HeLa cells were lysed using dounce homogenization and spun down at 700 × *g* for 10 min at 4°C. The supernatant containing cytosolic and mitochondrial fractions was further spun at 3,000 × *g* for 15 min at 4°C to minimize lysosomal and peroxisomal contamination. Finally, the pellet was rinsed in buffer with a 12,000 × *g* spin for 5 min and resuspended in PBS or complement assay buffer (25 mM HEPES [pH 7.4], 145 mM NaCl, 0.5 mM MgCl2, and 0.15 mM CaCl2) (58) for downstream analysis. Mitochondrial isolates were mixed 1:1 with anti-TOM20 antibody (20 µg/mL) and allowed to complex at room temperature for 1 h. Serum was diluted to a final concentration of 1 in 60 and added to the complexed mitochondrial mixture. Complement activation assays were performed in 100 μL reactions at 37 °C for indicated times and subjected to Western blot analysis.

### *In vitro* cleavage assay

*In vitro* cleavage assay was performed as previously described (59, 60). Briefly, HeLa cell lysate (30 µg) was incubated with purified wild-type CVB3 proteinase 3C (0.1 µg) or 2A (0.3 µg) in a cleavage assay buffer (20 mM HEPES, pH 7.4, 150 mM potassium acetate, and 1 mM dithiothreitol [DTT]) for 16 h at 37°C. Reaction was terminated with 6× SDS sample buffer, followed by 95°C denaturation and subsequent Western blot analysis.

### RT-qPCR

Total RNA was extracted using the Monarch Total RNA Miniprep Kit (New England Biolabs, #T2010S). To determine gene expression levels, qPCR targeting the following select genes: *IFNB*, *IL6*, *CXCL10*, *CCL5*, *IL-1B*, and *TNFA* was performed in a 10 µL reaction containing 1 µg of RNA using the Luna Universal One-Step RT-qPCR kit (New England Biolabs, #E3005L) and normalized to *ACTB* mRNA according to the manufacturer’s instructions. The PCR reaction was performed on a ViiA 7 Real-Time PCR System (Applied Biosystems). Samples were run in biological triplicate and analyzed using the comparative CT (2−ΔΔCT) method with control samples, and results were presented as relative quantitation. Primer sequences for RT-qPCR are provided in the table below.

### Quantification and statistical analysis

Quantification of immunohistochemistry images was performed using ImageJ (version 1.54f). Images were subjected to color deconvolution followed by intensity measurement. Optical density (OD) was calculated as OD = log (max intensity / mean intensity), where the value of max intensity was 255. Measurements are representative of three cardiac tissue samples from murine or human origin, as indicated in the respective figure legends. Statistical analysis was performed using GraphPad Prism (version 5) or Microsoft Excel software. Statistical differences between the two groups were calculated using an unpaired Student *t*-test. Comparison of multiple groups was statistically analyzed with analysis of variance with Tukey post hoc test.

## Data Availability

The data generated in the current study are available from the corresponding author upon reasonable request. All other data are publicly available as described in the relevant Materials and Methods section.

## SUPPLEMENTAL MATERIAL

Supplemental figures jvi.01804-24-s0001.pdf Figures S1 to S4.

ASM does not own the copyrights to Supplemental Material that may be linked to, or accessed through, an article. The authors have granted ASM a non-exclusive, world-wide license to publish the Supplemental Material files. Please contact the corresponding author directly for reuse.
