# Supplementary material for: Mitochondrial injury and complement dysregulation are drivers of pathological inflammation in viral myocarditis
Source: J Virol. 2025 Jan 23;99(2):e01804-24. doi: 10.1128/jvi.01804-24 (PMC11852726; doi:10.1128/jvi.01804-24)
Supplement: Supplemental figures — Figures S1 to S4. [file jvi.01804-24-s0001.pdf]

Supplemental Figure 1.

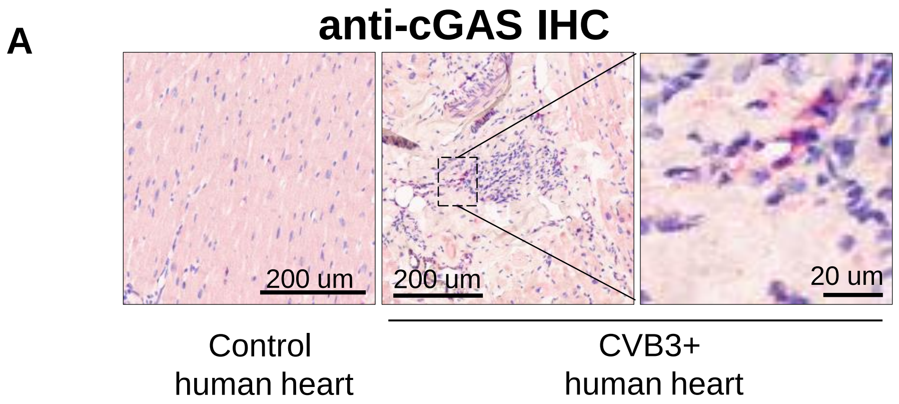

A. Immunohistochemistry of control and CVB3 (+) human heart tissue stained with anti-cGAS antibody. Inset is a zoom in of inflammatory cell infiltrates with positive staining for cGAS (red).

Supplemental Figure 2.

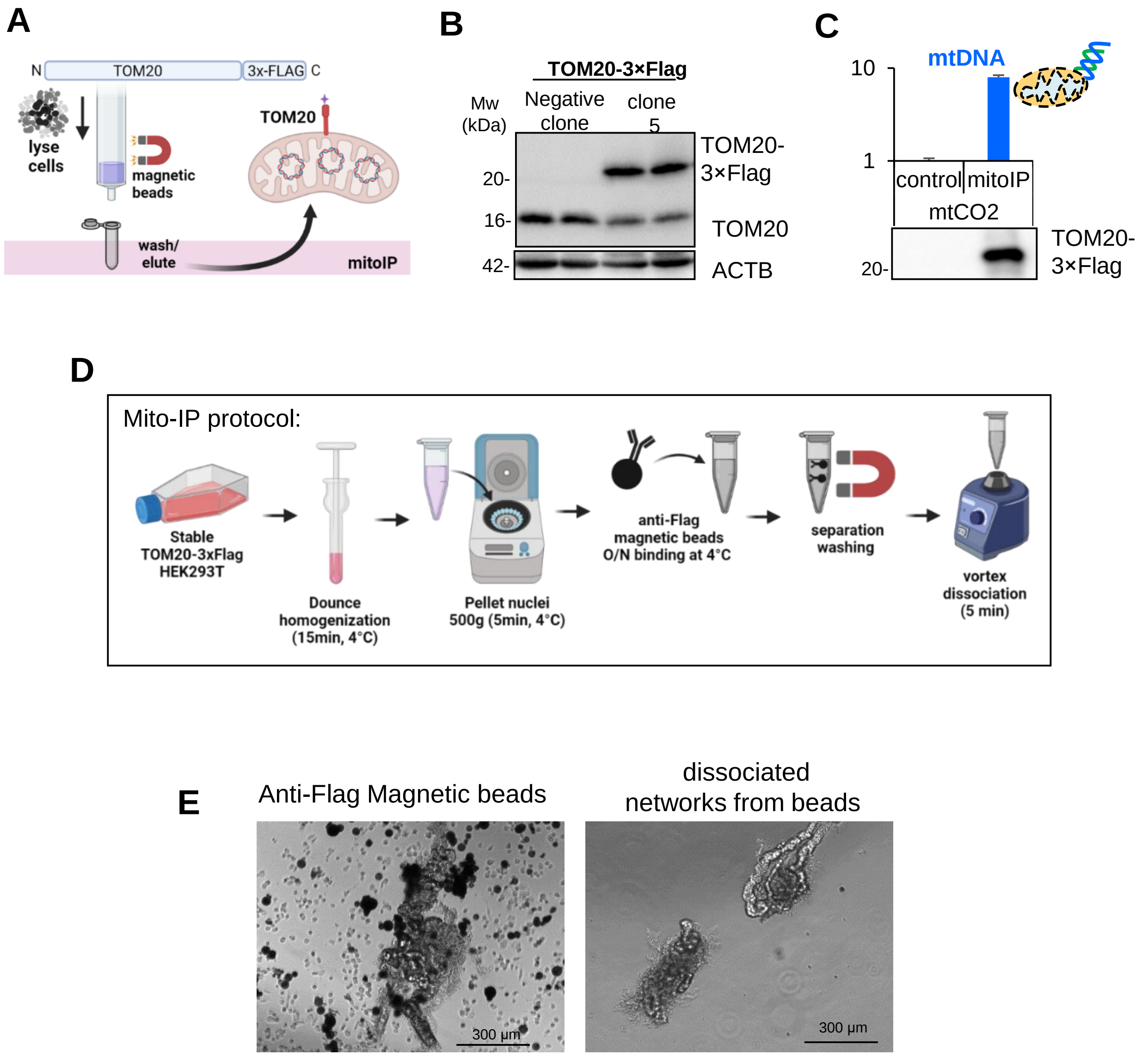

A. Schematic illustration of recombinant TOM20-3xFlag construct and simplified work flow for mito-IP
B. Generation of stable TOM20-3x-Flag expressing cells was validated with anti-TOM20 immuno-stain.
C. Validation of mitochondrial immunoprecipitation (mitoIP) with mtDNA marker mtCO2 enrichment and anti-TOM20 antibody

D. Schematic illustration of Mito-IP protocol
E. Bright field image of isolated and dissociated mitochondrial networks from mito-IP. Scale bar= 300 μm

Supplemental Figure 3.

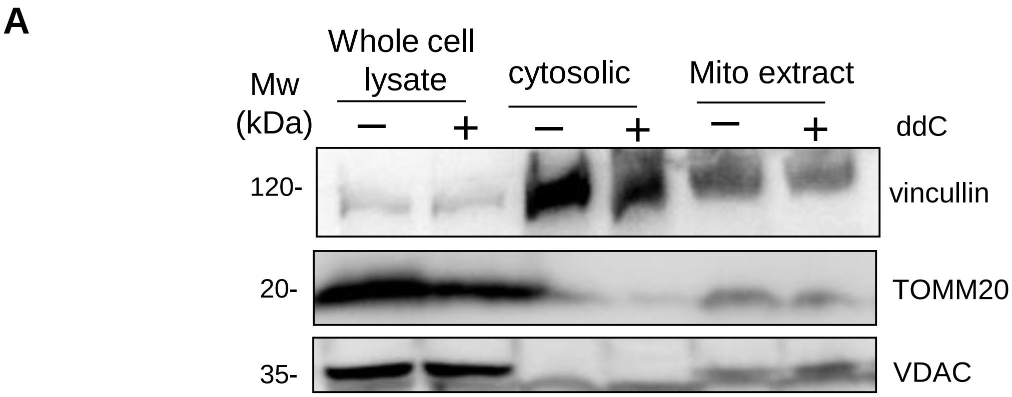

A. Cell fractionation of crude mitochondria extract treated with ddC (50ug/mL) or control was subjected to western analysis. Fractions of cytosolic or mitochondria compartments were assessed alongside whole cell lysate as control. Mitochondria enrichment was assessed with the presence of mitochondrial membrane proteins TOM20 and VDAC. Vincullin was immunostained as a predominantly cytosolic protein.

Supplemental Figure 4.

A

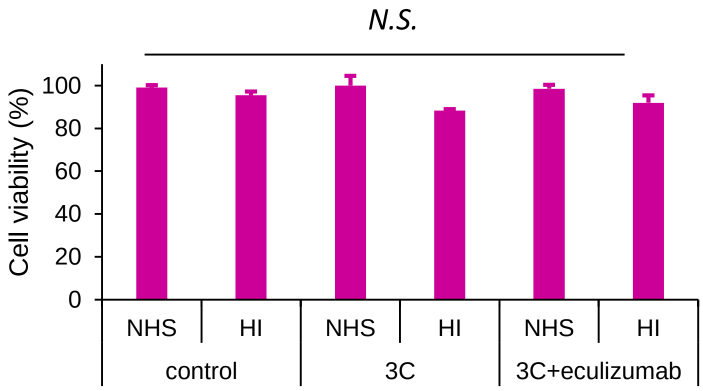

B

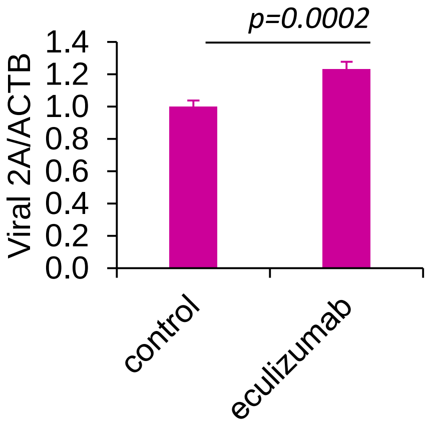

(A) Cell viability assay of HeLa cells incubated with either purified recombinant viral proteinase 3C (+/-) monoclonal anti-C5 antibody in the presence of either normal human serum (NHS) or heat-inactivated (HI) serum. Statistical analysis between groups was tested with ANOVA with Tukey post-hoc test (mean+/- S.D., n=3).

(B) HL1 mouse cardiomyocytes where infected with CVB3 (MOI=10, 24h) and total RNA was harvested for qPCR analysis of viral replication (2A viral protein) and normalized to ACTB housekeeping gene. Statistical analysis between groups was tested with unpaired student t test (mean+/- S.D., n=3).
